# Effect of the Home to Hospital to Home nutrition management program on postoperative liver cancer patients: A single-center randomized controlled study

**DOI:** 10.1097/MD.0000000000036648

**Published:** 2023-12-08

**Authors:** Runan Zhao, Xiaohong Hou, Yushuo Niu, Jianlu Wang

**Affiliations:** a Shandong Provincial Hospital Affiliated to Shandong First Medical University, Jinan, Shandong Province, China.

**Keywords:** Home to Hospital to Home, liver cancer, nutrition management, treatment outcome

## Abstract

**Background:**

Malnutrition is the most common complication in postoperative liver cancer patients. This study aimed to investigate the effects of the Home to Hospital to Home nutrition management program on postoperative liver cancer patients.

**Methods:**

A total of 184 liver cancer patients were randomly assigned to either an intervention group (treated under the Home to Hospital to Home nutrition management program) or a control group (treated with the general nutritional method). Nutrition-related indicators, such as prealbumin (PA) and albumin, were assessed before and after treatment in both groups. The incidence of complications (e.g., nausea or vomiting, bloating, difficulty defecating, ascites), length of hospital stay, first time to anal exhaust and out-of-bed activity, and patient satisfaction were recorded.

**Results:**

A total of 184 liver cancer patients were randomly assigned to either an intervention group (treated under the Home to Hospital to Home nutrition management program) or a control group (treated with the general nutritional method). Nutrition-related indicators, such as prealbumin (PA) and albumin, were assessed before and after treatment in both groups. The incidence of complications (e.g., nausea or vomiting, bloating, difficulty defecating, ascites), length of hospital stay, first time to anal exhaust and out-of-bed activity, and patient satisfaction were recorded.

**Conclusion:**

The Home to Hospital to Home nutrition management program improves the nutritional status of postoperative liver cancer patients, lowers the incidence of complications, shortens hospital stays, increases patient satisfaction, and promotes the early recovery of patients.

## 1. Introduction

Liver cancer is one of the most prevalent malignant tumors in China, and malnutrition is a severe complication following surgery.^[1]^ Moreover, the extensive nature of the surgical procedures and the high level of trauma lead to a decline in postoperative immune function and an increased catabolic state in the body, which significantly affects the postoperative recovery of patients.^[2]^ Nutritional support is considered a standard treatment for promoting liver regeneration after subtotal hepatectomy. It not only improves clinical outcomes for surgical patients but also reduces the incidence of complications and mortality.^[3,4]^

Enhanced recovery after surgery (ERAS) has gained widespread use across various disciplines, along with evolving concepts and methodologies. ERAS refers to the use of a series of optimized management measures with evidence-based medical evidence in the perioperative period to achieve the purpose of accelerating recovery, and it clearly states the significance of preoperative nutritional support.^[5]^ Pre-rehabilitation, a novel preoperative management strategy based on the principles of ERAS, aims to enhance patients’ physiological reserves, improving their ability to withstand surgical stress and facilitating a quicker return to a functional state suitable for daily activities through continuous preoperative nursing interventions, such as nutrition and exercise.^[6–8]^ Pre-rehabilitation emphasizes the importance of preoperative nutritional support again. However, nutritional management for liver cancer mainly focuses on the post-admission period, with little attention given to the nutritional status of patients before admission. Ma et al found that inadequate food intake in cancer patients before surgery may result in immune dysfunction, impaired intestinal mucosal barriers, increased postoperative complications, and higher mortality rates.^[9]^ Therefore, there is an essential need to emphasize preoperative nutritional support.

The Hospital to Hospital to Home mode is a comprehensive support service that first proposed by West China Hospital of Sichuan University in China in 2013, with a focus on extends the nutritional treatment of patients from the hospital to the outside of the hospital, improves the quality of life of patients and reduces their readmission rate.^[10–12]^ Guided by the concept of pre-rehabilitation, we incorporated prehospital nutritional intervention into the existing Hospital-Home model, proposing the Home to Hospital to Home (3H) nutrition management program. The implementation of the 3H management program proceeded as follows: patients with nutritional risks for elective surgery were readmitted to the hospital after one week of home-based nutrition treatment for evaluation and surgical intervention. Perioperative nutrition treatment was conducted during the hospital stay, and the management mode of home-based nutrition therapy was continued after discharge. This study was aimed to investigate the effects of the 3H management program on postoperative liver cancer patients compared to those receiving routine care in a similar setting, focusing on nutritional status, length of hospital stay, and incidence of complications.

## 2. Materials and methods

Between March 2021 and December 2021, patients with liver cancer scheduled for operative therapy at Shandong Provincial Hospital in Jinan, Shandong, China were invited to participate in the study. The inclusion criteria for the study were as follows: (1) patients ranged in age above 18 years till 85 years old; (2) patient who confirmed that he have liver cancer by abdominal ultrasound, enhanced CT scan, and liver biopsy; (3) patients who were fit for surgery; (4) patients with no preoperative vital organ dysfunction (e.g., heart, brain, kidney disease); (5) patients with the absence of infectious comorbidities; (6) patients with informed consent provided by the patient and family; (7) patients with Child-Pugh grading of A to B. Participants who met any of the following criteria were excluded from the study: (1) patients who were unable to communicate with data collectors; (2) patients who failed to complete surgery due to various reasons; (3) patients who withdrew from the study.

**Figure 1.**
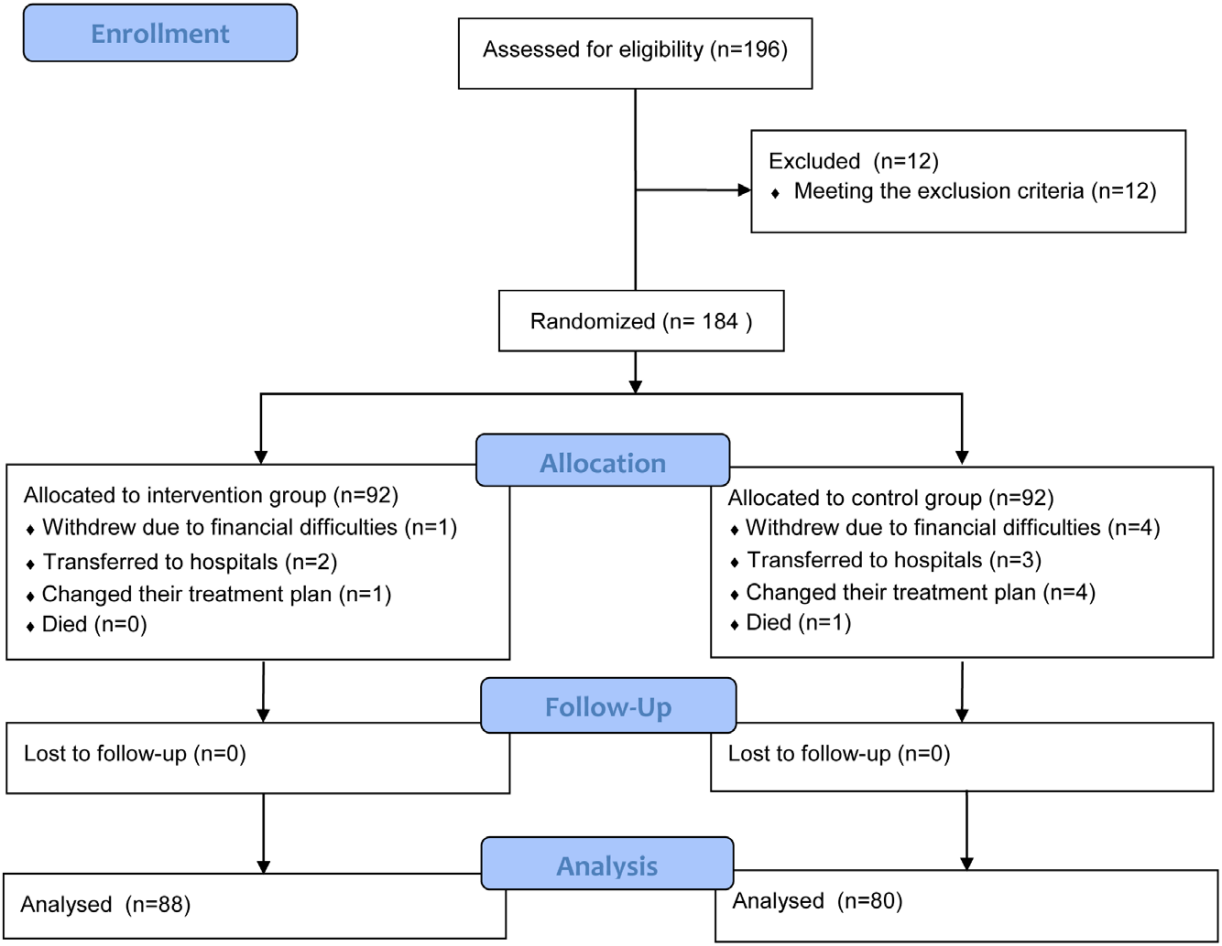
Patient flow chart including the number of screenings performed, the number of participants enrolled, the number of patients who dropped out, and final number of patients.

Patients were assigned numbers based on their order of admission and randomly allocated to either the intervention or control group. The allocation sequence was generated using blinded envelopes prepared by a caregiver with no knowledge of the trial. The envelopes contained an equal number of intervention and control protocols. Patients were assigned in the order of enrollment, and the researchers involved in recruitment, data collection, and analysis were blind to the allocation process. Sample size calculation was performed using PASS software 2020 (NCSS LLC., Kaysville, UT, USA).^[13]^ Data from a previous study were utilized, with serum albumin (ALB) being the primary outcome measure.^[14]^ In this study, a power of 90%, alpha of 0.025, mean of 41.5 in the intervention group, mean of 39.7 in the control group, and standard deviation of 4.5 were considered. The sample sizes of the 2 groups were equal, with a ratio of 1:1. The sample size for the case group was determined to be 157. An additional refusal rate of 10% to 20% was taken into account. Consequently, a total of 184 eligible patients who met the criteria and provided informed consent were recruited, while 16 patients were excluded. Finally, 168 participants (88 in the intervention group and 80 in the control group) were included in the study (Fig. 1). Ethical approval for the study was obtained from the Ethics Committee of Shandong Provincial Hospital (SWYX: No. 2023-096).

### 2.1. Intervention

In this study, all patients received different enteral nutrition interventions, while all other procedures remained the same. The control group received routine care, which included nutritional risk screening (NRS2002) by the responsible nurse upon admission and dietary education for patients with nutritional risk. The Nutritional Risk Screening is a valid and reliable tool used to assess the risk of malnutrition in hospital patients.^[15]^ It mainly includes nutritional status, disease severity and age, and the total score of ≥3 is classified as having nutritional risk. Preoperative preparation or nutritional intervention was adjusted according to each patient’s condition. Patients were instructed to consume water and liquid diets based on their tolerance (e.g., rice soup, fish soup, egg soup, porridge, milk). At the time of discharge, they were given oral instructions about the importance of a nutritious diet, and regular telephone follow-ups were conducted.

The research group implemented a 3H nutrition management program, guided by the documents explored in Nutrition Support Team (NST) and the current practice guidelines for nutritional care of liver cancer patients. Thirteen clinical nursing specialists and nutrition specialists were consulted to develop the final intervention protocol. The specific steps of the program were as follows:

Step 1: NST establishment. The team consisted of 2 physicians, 2 dietitians, 1 pharmacist, 2 experienced nurses (who had been in charge of nurses and had more than 5 years of work experience), and 1 core member from the hospital’s nutrition academic team. All team members are employed by Shandong Provincial Hospital. The roles of the NST included NRS2002, regular monitoring of nutritional status, formulation and modification of nutritional treatment plans, placement and maintenance of nutritional treatment pathways (e.g., gastric tubes, nutritional tubes), monitoring and treatment of patient complications and adverse reactions, dietary guidance, education, research development, and continuous quality improvement. Each team member had assigned responsibilities, but there was collaboration and communication among them.

Step 2: Home intervention. Patients expected to undergo liver cancer surgery were assessed for nutritional risk using the NRS2002 tool during consultation with nutritionists at the outpatient department. Patients with no nutritional risk were admitted to the hospital for elective surgery. For patients with a nutritional risk, dietary plans were developed by the NST based on their nutritional status. These plans primarily consisted of oral nutritional supplements (ONS). ONS is formulated as food for specific medical purposes, typically containing a balanced blend of macronutrients (e.g., protein, carbohydrates, and fats) as well as micronutrients (e.g., vitamins and minerals), which provide balanced nutrients to meet the needs of the body for nutrients.^[16]^ In addition to 3 regular meals, patients received 250 to 300 mL of ONS orally at 9:30, 15:00, and 21:30 daily. A WeChat group was used for daily follow-up and evaluation. After 7 days of home-based nutritional treatment, the patient was admitted to the hospital.

Step 3: Hospital intervention. Upon admission, the nurse conducted NRS2002 and provided dietary education. Preoperative patients were recommended a light, easily digestible liquid and semiliquid diet. The target intake of energy is 25 to 30 kcal/(kg·d), and the target protein is 1.2 to 1.5 g/(kg·d). Carbohydrate intake was given in separate oral doses of 355 mL of 12.5% solution between 5 a.m. and 6 a.m. on the day of the operation, with an additional 355 mL administered 2 to 4 hours before surgery in divided doses. After 6 hours of being awake following anesthesia, water intake could begin with approximately 20 mL, followed by 50 to 100 mL every 2 to 3 hours. A liquid diet was started 24 hours after surgery, and 100 to 150 mL of ONS was given every 2 to 3 hours, aiming for a total intake of 500 to 750 mL that day. The intake was gradually increased by 50 ml every 2 to 3 hours based on patient tolerance. After 48 to 72 hours, a semiliquid diet was resumed. Once anal exhaust occurred, patients resumed a normal diet. The NST collaborated with the nutrition department to develop personalized diets for patients and established a dietary activity record table. Regular nutrition-related lectures were held by the nutrition team to improve compliance among patients and their families, explain the importance of enteral nutrition, address dietary misconceptions, and answer questions. Many patients and their families have dietary misconceptions. For example, some patients and their families may be influenced by traditional Chinese beliefs that consumption of fish and shrimp possesses stimulating properties, which may hinder wound healing. Nevertheless, fish and shrimp possess a wealth of high-quality protein and a diverse range of trace elements that are easily absorbed and utilized by the human body. Similarly, patients often experience a pronounced thirst and hunger following emergence from anesthesia, which is exacerbated by heightened levels of anxiety expressed by their loved ones. Consequently, premature administration of excessive postoperative supplements may arise, resulting in symptoms such as abdominal distension, nausea, and vomiting.

Step 4: Home intervention. On the day of discharge, patients were reassessed for nutritional risk. Nutritional therapy continued after discharge for patients at risk of malnutrition or those who required perioperative intervention but did not meet their energy needs through oral routes. The therapy primarily involved ONS, which were added 3 times a day in addition to regular meals.

Step 5: Follow-up. A comprehensive doctor-nurse-dietitian follow-up support system was established, and a WeChat group was created. The patients were monitored for a duration of 2 weeks post-discharge. Patients were instructed to maintain a diet diary, recording food intake information for each meal and regularly uploading it once a day. Team members reviewed the information daily and provided personalized guidance as needed. Patients had a follow-up review 2 weeks after discharge.

### 2.2. Observation indicators

1. The ALB: It is primarily synthesized in the human liver and serves to maintain colloid osmotic pressure and transport many exogenous and endogenous substances, which reflects the nutritional status of the body as well as the severity of underlying diseases.
2. Prealbumin (PA): it is a transportation protein that is primarily present in the bloodstream and has greater sensitivity than albumin when identifying sudden fluctuations in proteins. From a clinical standpoint, it is frequently employed as an indicator to gauge liver function impairment and different forms of malnourishment.

### 2.3. Secondary outcome measures

1. The first time to anal exhaust and out of bed activity: the first time to anal exhaust and out of bed activity since returning to the ward after surgery.
2. The average length of hospital stay: the average length of hospital stay for each patient in a certain period. The average length of stay = total bed days occupied by discharged patients/number of discharged patients in the same period.
3. Postoperative complications: the number of patients with complications, including nausea or vomiting, bloating, difficulty defecating, and ascites, compared between postoperative levels and at discharge.
4. The satisfaction of patients: the satisfaction level of patients was measured using the nursing satisfaction rating scale questionnaire, designed by Shandong Provincial Hospital, 2 weeks after discharge. This instrument includes 9 items. Items are scored on a five-point Likert scale, ranging from 1 (very dissatisfied) to 5 (very satisfied). The questionnaire demonstrated an acceptable level of internal consistency with Cronbach alpha value of 0.947.

### 2.4. Additional information

The age, sex, BMI, marital status, degree of education, diameter of the tumor, and child grading of liver function of the patients were also recorded using the hospital information system.

The following measures were taken to ensure the validity and reliability of the result measurement: (1) strict adherence to exclusion criteria when selecting eligible research participants; (2) recruitment of questionnaire investigators who were qualified nurses working in the same hospital’s hepatobiliary surgery ward and had undergone standardized training; (3) immediate scrutiny of completed questionnaires by investigators to eliminate any invalid ones; (4) implementation of a two-person data entry method into Excel 2019 to establish a database, ensuring input data accuracy and integrity; (5) inclusion of statisticians with relevant statistical knowledge background.

### 2.5. Statistical analysis

SPSS version 21.0 (IBM Corp., Armonk, NY) was used to perform all statistical analyses.^[17]^ Continuous variables that followed a normal distribution, such as age, BMI, and other measurements, are presented as mean ± standard deviation (SD), and *t*-tests were used for group comparisons. Categorical variables, such as sex and marital status, were presented as frequency and percentage, and chi-squared tests or Fisher exact tests were applied for data comparison. All analyses were performed at a significance level of *P* < .05.

## 3. Results

### 3.1. Baseline characteristics of patients

The study framework is shown in Figure 1. A total of 184 patients with liver cancer were assessed for eligibility, and eventually, 168 of them were enrolled. Participants were randomly assigned to either the intervention group (n = 92) or the control group (n = 92). During the intervention, 5 patients withdrew due to financial difficulties (1 in the intervention group, 4 in the control group), 5 patients were transferred to different hospitals (2 in the intervention group, 3 in the control group), and 5 patients changed their treatment plan (1 in the intervention group, 4 in the control group). Additionally, one patient in the control group died. Finally, the control group consisted of 56 men and 24 women, with an average age of 56.84 ± 1.07. The intervention group consisted of 60 men and 28 women, with an average age of 57.31 ± 0.99. As shown in Table 1, the baseline characteristics of the 2 groups were similar in terms of age, sex, marital status, education, tumor diameter, and Child-Pugh grade, with no statistical differences between the 2 groups (*P* > .05).

**Table 1:** Characteristics of the study sample.

Characteristics		Experimental group (n = 88)	Control group (n = 80)	*t/χ^2^* value*	*P* value*
Age (years) (mean ± SD)		57.31 ± 0.99	56.84 ± 1.07	0.923*	>.05
Sex, male/female, n (%)	Male	60 (68.2)	56 (70.0)	0.799†	>.05
	Female	28 (31.8)	24 (30.0)		
Marital status, n (%)	Married	84 (95.5)	74 (92.5)	0.692†	>.05
	Divorced	1 (1.1)	2 (2.5)		
	Bereft of one’s spouse	3 (3.4)	4 (5.0)		
BMI (mean ± SD)		25.07 ± 3.43	23.67 ± 3.20	0.161*	>.05
Education level, n (%)	Bachelor degree or above	4 (4.5)	4 (5.0)	0.95†	>.05
	High school/technical secondary school	27 (30.7)	29 (36.3)		
	Junior high	36 (40.9)	30 (37.5)		
	Primary school	18 (20.5)	15 (18.8)		
	Illiteracy	3 (3.4)	2 (2.5)		
Diameter of tumor		4.46 ± 0.36	4.36 ± 0.48	1.597*	>.05
Child-Pugh, n (%)	A	61 (69.3)	50 (62.5)	0.350†	>.05
	B	27 (30.7)	30 (37.5)		

BMI = body mass index;

* Derived from *t*-tests on data.

† Derived from Chi-squared tests on data.

### 3.2. Nutritional status

As shown in Table 2, the levels of PA and ALB in the intervention group were higher than those in the control group after implementing different methods of nutrition management, and this difference was statistically significant (*P* < .05).

**Table 2:** Comparison of nutrition-related indicators between the control and intervention groups before and after nutritional intervention.

Nutrition-related indicators		Experimental group (n = 88)	Control group (n = 80)	*P* value*
Albumin (g/L)	Baseline	33.37 ± 3.22	32.87 ± 3.66	.34
	Admission	38.06 ± 2.52	32.63 ± 2.99	<.001
	Ending	35.03 ± 2.65	33.98 ± 3.53	.03
Prealbumin (g/L)	Baseline	143.55 ± 53.88	141.47 ± 52.01	.80
	Admission	218.16 ± 57.21	151.59 ± 60.41	<.001
	Ending	141.84 ± 52.02	93.34 ± 39.81	<.001

* *P* values were derived from *t* tests on data.

### 3.3. Incidence of complications and clinical outcomes

In the intervention group, the number of cases with postoperative abdominal distension, defecation difficulty, and ascites was lower compared to the control group, and these differences were statistically significant (*P* < .05) (Table 3). The incidence of postoperative nausea and vomiting was also lower in the intervention group, although the differences between the 2 groups were not statistically significant (*P* > .05) (Table 3).

**Table 3:** Comparison of secondary outcome indicators between the intervention and control groups.

Measurement index	Experimental group (n = 88)	Control group (n = 80)	*P* value
The first time to anal exhaust (h)	45.49 ± 8.58	66.81 ± 8.87	<.001*
The first time to get out of bed activity (h)	33.47 ± 7.38	68.89 ± 9.41	<.001*
The average length of hospital stay (d)	6.27 ± 1.03	10.75 ± 2.50	<.001*
Number of patients with postoperative complications			
Nausea or vomiting, n (%)	12 (13.6)	17 (21.3)	.20†
Bloating, n (%)	7 (8.0)	15 (18.8)	.038†
Difficulty defecating	4 (4.5)	15 (18.8)	.006†
Ascite, n (%)	2 (2.3)	9 (11.3)	.019†
The satisfaction of patients	99. 54 ± 0.61	96. 20 ± 1.69	<.001*

* Derived from *t* tests on data.

† Derived from Chi-squared tests on data.

Regarding clinical outcomes, Table 3 demonstrates that the time to first anal exhaust and out-of-bed activity were statistically significant (*P* < .01). Additionally, the length of hospital stay was recorded for both groups and was lower in the observation group compared to the control group, with a significant difference in the average duration of hospital stay between the 2 groups (*P* < .01).

### 3.4. The satisfaction of patients

Table 3 presents the mean scores and standard deviation of the nursing satisfaction rating scale questionnaire. The total score of patient satisfaction was significantly different between the 2 groups (*P* < .05), with the intervention group reporting significantly higher satisfaction compared to the control group (*P* < .05).

## 4. Discussion

In China, the role of preoperative nutritional support for patients with liver cancer has not been adequately acknowledged and emphasized. The present study evaluates the impact of nutritional support on liver cancer patients during and after surgical intervention. The aim of the study is to investigate the effects of implementing a comprehensive 3H management program on the nutritional status of patients with gastrointestinal malignancies. The findings of the study indicate that the implementation of a 3H nutrition management program significantly improves the nutritional status of cancer patients, resulting in the restoration of intestinal function, shorter duration of hospitalization, and reduced postoperative complications.

ALB and PA levels are commonly used to monitor the nutritional status of the body, reflecting long-term and short-term nutritional statuses, respectively.^[18]^ The ALB and PA levels in the observation group were significantly higher than those in the control group, suggesting that the 3H nutrition management program effectively enhances the postoperative nutritional status of patients. Improved nutritional indicators were associated with a significant reduction in hospital stay duration, consistent with the findings of this study.^[19]^ However, the study also revealed a significant decrease in nutritional indexes in both groups after the intervention period compared to baseline. This decline can be attributed to increased catabolism, protein anabolic resistance, reduced digestion and absorption function, and the inability to maintain a positive nitrogen balance following liver surgery, ultimately affecting the postoperative nutritional status of patients.^[20]^

Liver cancer surgery poses a high trauma and complication risk, often accompanied by a risk of malnutrition and general health deterioration.^[9,21]^ In a study on cholangiocarcinoma patients, an early enteral nutrition program was found to reduce the incidence of postoperative complications, improve clinical outcomes and immunological functions, thereby benefiting patient recovery.^[9]^ Reis et al discovered that adequate nutritional support accelerated the resolution of abdominal distension, reduced its severity, and facilitated intestinal function recovery.^[22]^ Similarly, the number of postoperative complications, including abdominal distension, difficulty in defecation, and ascites, was lower in the nutritional intervention group compared to the control group in the present study. This may be attributed to the activation of the intestinal neuroendocrine system, maintenance of normal immune system function, preservation of intestinal mucosal integrity, and the role played by nutritional support in gastrointestinal function recovery.^[23,24]^ Unexpectedly, although the number of cases with postoperative nausea and vomiting was lower in the intervention group than in the control group, there was no statistical significance between the 2 groups. This may be related to the various causes of nausea and vomiting, such as increased vagal tone after epidural anesthesia, postoperative opioid use, and laparoscopic surgery.^[25]^

Moreover, it can significantly reduce the time to primary anal exhaust and out-of-bed activity. Similar to this study, Wang et al implemented gastrointestinal management under the concept of accelerated rehabilitation for liver cancer, which ultimately alleviated thirst, hunger, and other discomfort symptoms, promoted anal exhaust, and improved nutritional status.^[26]^ Anal exhaust time reflects the recovery of gastrointestinal function. In this study, we provided patients with a comprehensive refined nutrition management approach, developed personalized diet recipes, established dietary activity records after accelerated rehabilitation surgery, and thus facilitated the recovery of gastrointestinal function. Additionally, early resumption of feeding can reduce the release of inflammatory mediators, promote body function recovery, and facilitate earlier mobilization.^[27]^ Early postoperative activity is vital in the context of ERAS for patients undergoing liver surgery, as it helps reduce the adverse effects of prolonged bed rest.^[28]^ Furthermore, it reduces the incidence of pulmonary complications, thromboembolic diseases, and insulin resistance, thus promoting early patient recovery.^[29,30]^

The study demonstrated that the satisfaction score of patients in the observation group was higher than that of the control group. The ONS nutrition program was implemented prior to admission, and nursing intervention was strengthened to meet the duration of nutritional support required by patients before surgery as much as possible. During the perioperative period, NST provided professional and standardized dietary guidance to patients. Additionally, nutrition intervention programs based on ONS were developed for post-discharge patients, and follow-up support systems integrating the expertise of doctors, nurses, and dietitians were established. Simultaneously, the WeChat platform was utilized for personalized nutrition guidance combined with an evaluation of the patient’s family situation. The NST dynamically considered the nutritional treatment of patients outside the hospital and guided patients during self-monitoring and management. Overall, the 3H nutrition management program allowed patients to leave the hospital without concerns, increased trust between healthcare providers and patients, and consequently improved patient satisfaction with nursing care.

Partial hepatectomy is the most common treatment method for liver cancer.^[31]^ However, it can induce stress on the body and reduce liver function due to the necessity of blocking the hepatic hilum during surgery, which may lead to postoperative malnutrition and delayed recovery of patients.^[3,32]^ Therefore, nutritional support plays a crucial role in managing and rehabilitating patients undergoing hepatectomy. The 3H nutrition management program emphasizes the integration of nutritional support before admission, during the perioperative period, and after discharge. To our knowledge, this is the first study to add nutritional support prior to admission with liver cancer in China. The results of this research offer significant insights for healthcare professionals, especially oncologists and nutritionists, who can implement nutritional assistance and intervention programs for individuals with cancer in China. Such interventions will be instrumental in enhancing patient outcomes.

### 4.1. Study limitations

There were several limitations in our study that should be acknowledged. First, it was a single-center report with a limited number of patients. Second, it was a randomized controlled single-blind trial, and due to the nature of the intervention, doctors and nurses could not be blinded. Third, we only collected data after the intervention and failed to collect data at multiple time points during the intervention, thereby preventing us from determining the dynamic changes in outcome indicators. Finally, PA and ALB were primary indicators of nutritional status in this study. However, more detailed nutrition-related or long-term indicators such as body composition and survival rates were not measured.

## 5. Conclusion

Our data suggest that the 3H nutrition management program was beneficial for improving the nutritional status of postoperative liver cancer patients, promoting rapid recovery of postoperative intestinal function, facilitating early ambulation, and enabling early patient recovery. Moreover, the 3H nutrition management program was a comprehensive and precise nutrition management model that reduced the likelihood of complications, improved clinical outcomes, and shortened hospital stays. Additionally, the collaboration of multidisciplinary teams facilitated communication between departments, enhanced trust between healthcare providers and patients, and improved patient satisfaction. In the future, additional intervention time points can be designed to observe dynamic changes in nutritional indices in patients, and further large-scale and long-term studies are needed to assess the long-term effects of the 3H nutrition management program.

## Acknowledgments

The participation of the patients and their families is gratefully acknowledged. We also thank the NST members of Shandong Province Hospital for their hard work.

## Author contributions

**Conceptualization:** Runan Zhao, Xiaohong Hou.

**Data curation:** Runan Zhao, Xiaohong Hou.

**Formal analysis:** Runan Zhao, Xiaohong Hou.

**Investigation:** Runan Zhao, Xiaohong Hou.

**Methodology:** Runan Zhao, Xiaohong Hou.

**Project administration:** Xiaohong Hou.

**Software:** Runan Zhao, Xiaohong Hou, Jianlu Wang.

**Supervision:** Niu Yushuo.

**Validation:** Niu Yushuo.

**Visualization:** Runan Zhao, Jianlu Wang.

**Writing – original draft:** Jianlu Wang, Niu Yushuo.

**Writing – review & editing:** Jianlu Wang, Niu Yushuo.
